# Fractal Dimension and Vessel Complexity in Patients with Cerebral Arteriovenous Malformations

**DOI:** 10.1371/journal.pone.0041148

**Published:** 2012-07-18

**Authors:** Gernot Reishofer, Karl Koschutnig, Christian Enzinger, Franz Ebner, Helmut Ahammer

**Affiliations:** 1 Department of Radiology, MR-Physics, Medical University of Graz, Graz, Austria; 2 Division of Neuroradiology, Department of Radiology, Medical University of Graz, Graz, Austria; 3 Department of Psychology, University of Graz, Graz, Austria; 4 Department of Neurology, Medical University of Graz, Graz, Austria; 5 Institute of Biophysics, Medical University of Graz, Graz, Austria; University of Jaén, Spain

## Abstract

The fractal dimension (*FD*) can be used as a measure for morphological complexity in biological systems. The aim of this study was to test the usefulness of this quantitative parameter in the context of cerebral vascular complexity. Fractal analysis was applied on ten patients with cerebral arteriovenous malformations (AVM) and ten healthy controls. Maximum intensity projections from Time-of-Flight MRI scans were analyzed using different measurements of *FD*, the Box-counting dimension, the Minkowski dimension and generalized dimensions evaluated by means of multifractal analysis. The physiological significance of this parameter was investigated by comparing values of *FD* first, with the maximum slope of contrast media transit obtained from dynamic contrast-enhanced MRI data and second, with the nidus size obtained from X-ray angiography data. We found that for all methods, the Box-counting dimension, the Minkowski dimension and the generalized dimensions *FD* was significantly higher in the hemisphere with AVM compared to the hemisphere without AVM indicating that *FD* is a sensitive parameter to capture vascular complexity. Furthermore we found a high correlation between *FD* and the maximum slope of contrast media transit and between *FD* and the size of the central nidus pointing out the physiological relevance of *FD*. The proposed method may therefore serve as an additional objective parameter, which can be assessed automatically and might assist in the complex workup of AVMs.

## Introduction

Cerebral arteriovenous malformations (AVMs) are defined by arteriovenous shunting through a nidus of coiled and tortuous vessels, that directly connect feeding arteries to draining veins [1]. Due to the increased number of vessels, the vascular network shows a higher structural complexity. Given that AVMs imply the risk of morbidity or mortality due to intracerebral hemorrhage or seizure, AVMs are usually treated after their diagnosis. Depending on size and location, various methods have been developed for clinical treatment including microsurgical resection, endovascular embolization and stereotactic radiosurgery such as Gamma Knife surgery [2], [3], [4].

Advances in magnetic resonance angiography (MRA) have improved the accuracy of revealing the structure and hemodynamics of AVMs, providing the basis for the choice of an optimal therapeutic approach. While 3D-Time-of-Flight (TOF) techniques are usually applied as non-invasive means of diagnosis to achieve high resolution spatial imaging of the vascular system [5], [6], dynamic contrast enhanced MRI (DCE-MRI) gives information about the vascular flow. Postprocessing methods such as the Volume Rendering technique and Maximum Intensity Projection (MIP) allow for a 2D representation of the blood vessels from different projections, providing a good overview about the vascular network. Usually AVMs are evaluated through experienced neuroradiologists, supported by multimodal imaging techniques. Structural and dynamic properties of the AVM provide the basis for diagnosis and further intervention. This difficult diagnostic process is user dependent and affected by inter-observer variation. An objective measure that is may be related to the underlying physiology of the AVM such as the enhanced vascular complexity of AVMs due to feeding arteries, contrast media transit and nidus size is expected to aid in classifying this complex vascular disease.

In the 1960s Benoit Mandelbrot introduced the term “fractal” for complex objects whose geometry cannot be characterized by an integral dimension. The measured metric properties (length, area or volume) of such a fractal object are a function of the scale of measurement. Considering the “classical” example of measuring the length of a coastline, the relationship between the measured metric and the scale of measurement is obvious [7]. This relationship is reflected in the fractal dimension (*FD*) of an object. A fractal object can be defined as an object, whose *FD* is greater than its topological or Euclidian dimension which is zero for a point, one for a curve and two for a plane. With this, the *FD* can describe many natural geometrical features such as self-similarity in textures or structures obtained by stochastic processes. In biophysical sciences *FD* is widely used as a tool to quantify the geometrical complexity of a structure by means of its space filling properties [8]. Various theories have been proposed to describe the *FD* of an object and numerical techniques have been developed to estimate the dimension of a fractal or as an extension to estimate generalized fractal dimensions obtained by multifractal analysis (MFA) [9], [10], [11].

Images representing morphologically complex structures can be described by means of the fractal dimension using fractal analysis. In the last few years, this image analysis technique raised attention in analyzing the shape and complexity of natural structures [8], [12], [13], [14], [15], [16], [17], [18]. Widespread applications of *FD* can be found in the field of neuroscience [19]. Since Kiselev et al. gave a positive answer to the question “Is the brain cortex a fractal?” [20], many attempts have been made to analyze the fractal structure of the brain [21], [22]. Various studies demonstrated that changes of the fractal dimension in the brain could be associated with neurological diseases such as Multiple Sclerosis [23], [24], Schizophrenia [25], and Alzheimer’s disease [26].

However, disorders in the human cerebral vascular system have not been investigated so far by means of fractal analysis. The fractal nature of vascular systems has been shown for the vascular tree in the human retina [27], for the vasculature of the human placenta [13] and for the pial vasculature in cats [28]. Supported by theoretical considerations about the fractality of tree like structures in biological systems, models of the cerebral vascular system have been developed based on fractal theory [29], [30].

This is a proof of concept study to investigate the influence of vascular complexity on values of *FD* and its relation to the underlying physiology in patients suffering from AVM. Based on skeletonized MIP images from 3D-TOF MRI data, we quantified the vascular complexity by means of *FD* using the widely used Box-counting dimension (*D_b_*) [31] and as a second method to assess *FD*, the Minkowski dimension (*D_m_*) [32]. If the structure to be analyzed is complex with subsets of regions that obey different scaling rules, a multifractal approach using generalized fractal dimensions may provide more information. Giving the fact, that natural structures can not be considered as perfect fractal (or monofractal) the fractal nature of the object can be characterized by a hierarchy of exponents [33]. We studied multifractal properties by applying MFA providing a generalized dimension spectrum. From this spectrum the capacity dimension (*D_0_*), the information dimension (*D_1_*) and the correlation dimension (*D_2_*) [34] were used for numerical analysis. All methods were applied and compared for ten patients with AVM and ten healthy controls. Considering, that *D_b_* depends on the image matrix size we additionally investigated this influence for in vivo data and for stochastically generated fractals obtained by diffusion limited aggregation (DLA) [35]. The physiological significance of *FD* was probed using regression analysis to reveal the correlation between *FD* and the maximum slope of contrast media transit from DSC-MRI data and the mean nidus size obtained from X-ray angiography data respectively.

## Materials and Methods

### Theory

The fractal dimension can be described theoretically by the Hausdorff dimension [36]. Giving the fact that the Hausdorff dimension can not be directly assessed, an approximation is possible, using the concept of self-similarity. Consider an object made up of distinct segments. If each segment is divided into *r* smaller segments, the resulting number *N* of smaller objects follows a power low:
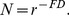
(1)


Hence,
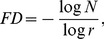
(2)where FD is the dimension of the scaling law. For Euclidean objects FD equals the Euclidean dimension (D = 1,2,3,…n). Fractal objects obey a metric scaling relation, where the exponent (the fractal dimension, FD) is not equal to the Euclidean dimension and is usually noninteger.

The Box-counting dimension [37] approximates the FD by covering a binary image with a grid of boxes of a length ε and counting the number of nonempty boxes *N*(ε). Starting with a single box covering the whole image, progressively the box length ε is reduced by a factor of two at each step and the corresponding numbers of nonempty boxes *N*(ε) are counted. *D_b_* is determined using the relationship:


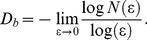
(3)

Linear regression can be performed to obtain the slope of the double logarithmic plot *log N(*ε*)* versus *log (*ε*)* providing *D_b_*.

The Minkowski or Minkowski-Bouligand dimension *D_m_* is given by:
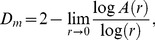
(4)where A(r) are the number of disks with the radius r, covering the object in the binary image. A relationship between the increasing radii and the area covering the object is given, similar to the Box-counting dimension, in the double logarithmic plot log A(r) versus log (r). The slope, assessed by linear regression gives Dm. A derivation of Dm can be found elsewhere [38].

Geometrical multifractals can be characterized in terms of their generalized dimensions D_q_ [39]. D_q_ of a binary image with M_0_ pixel and size L that is covered with a grid of boxes of size l is given by:
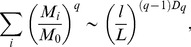
(5)where M_i_ is the number of pixel within the i-th box and q is variable within the limits 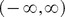. MFA provides a generalized dimension spectrum where for q = 0: *D_0_* is usually referred to as the capacity dimension, for q = 1: *D_1_* the information dimension and for q = 2: *D_2_* the correlation dimension respectively. These dimensions are related by the inequality



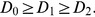
(6)Whereas D_q_ is independent from q for a monofractal structure, a geometric object can be considered as a multifractal if the inequality is fulfilled with statistically significant differences [40].

### Participants

Ten patients (P) (seven male, three female, mean age 44.1±18.5 years) showing a unilateral, supratentorial AVM with no acute bleeding (8 left hemisphere, 2 right hemisphere), which was diagnosed by an experienced neuroradiologist, and ten healthy controls (HC), four male, six female (mean age 51.4±13.1 years) showing no cerebral vascular abnormalities in the MRI scan, were included in this study. All patients and controls gave written informed consent to undergo the MRI protocol as specified below, and the study was approved by the local ethics committee of the Medical University of Graz.

### Magnetic Resonance Imaging

The MRI protocol included standard T_1_-, and T_2_-weighted sequences, a 3D-TOF sequence and a DCE-MRI sequence. 3D-TOF MR angiograms of the circle of Willis and vertebro-basilar arteries were obtained with the following parameters to visualize AVM: TR = 22 ms, TE = 3.68 ms, flip angle = 18°, FOV = 200 mm, phase FOV = 75%, image matrix = 384×288, number of slabs = 3, slices/slab = 52; slice thickness = 0.65 mm. This resulted in a spatial resolution of 0.52 mm×0.52 mm in plane. DCE-MRI data were acquired using a 3D-FLASH sequence with the following parameters: TR = 2.67 ms, TE = 1.05 ms, flip angle = 16°, FOV = 230 mm, image matrix = 320×320, number of slabs = 1, slices/slab = 12; slice thickness = 6 mm. A dose of 0.2 ml/kg body weight contrast agent (ProHance®, Bracco Diagnostics, Inc., Princeton, NJ, USA) was injected intravenously via a power injector (Spectris; Medrad Inc., Indianola, PA, USA) at a flow rate of 3 ml/s. All measurements were carried out on a 3T Tim Trio system (Siemens Medical Systems, Erlangen, Germany) using a 12 channel head coil.

### Image Processing

**Figure 1:**
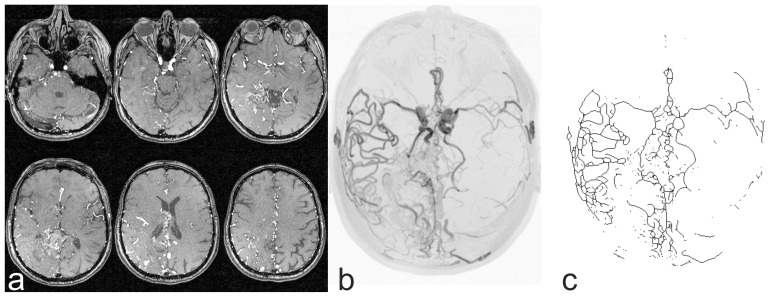
3D-TOF images. (a) provide the basis for MIP images (b). After segmentation and skeletonizing, binary images (c) are obtained that serve as input for the *FD* analysis. ((b) and (c) inverted view).

To minimize the effect of inter-subject variability on *FD* due to differences in head size and FOV positioning, the 3D-TOF images of each patient were spatially normalized into the standard MNI space (Montreal Neurological Institute) using FLIRT [41], a linear registration tool and part of the FMRIB software library (FMRIB Centre, University of Oxford, UK; http://www.fmrib.ox.ac.uk/fsl/fsl/downloading.html). An affine 12 parameter model was applied to coregister the 3D-TOF data sets to a template (MNI152_T1_0.5 mm) resulting in spatially normalized 3D data sets with a matrix size of 364×436×364 each. MIP was done for all patients and controls using MRIcro software (Chris Rorden, University of Nottingham, UK; http://www.nitrc.org/frs/download.php/414/mrizip.zip) and images of the axial projection (azimuth 0°, elevation 90°) were saved in the “TIF” image format. To capture the vascular tree and to remove the background from the MIP images, a k - means clustering algorithm was applied. Afterwards the images were converted into binary images and skeletonized (Figure 1) using the ImageJ software v.1.45 (Wayne Rasband, National Institutes of Health, USA; http://rsbweb.nih.gov/ij/download.html). The images from the patients and from the controls were separated into two halves with a resolution of 182×436 each to enable a statistical comparison between both hemispheres.

The maximum slope of contrast media transit was evaluated from DCE-MRI based MIP data. Intensity-time curves from the hemispheres containing the AVM were determined for each patient. The maximum slope is given by the maximum of the differentiated intensity-time curve. Analysis was done with software built in house using Matlab software (V 2010a,The MathWorks, Inc., MA, USA).

Selective X-ray contrast enhanced angiography was performed within three weeks from date of MRI scans for all patients to visualize the nidus in coronal and sagittal view. The nidus size was estimated by the average of the minimum diameter and the maximum diameter from both views to approximate the mean diameter.

### Fractal Dimension

In order to quantify the vessel complexity two methods were applied, using the relation (3) and (4), to access the fractal dimension: the Box-counting dimension and the Minkowski dimension.

For the evaluation of the Box-counting dimension, the size of the squares forming a grid to cover the binary image was given by ε = 2^n^, where n grew with increment one in each iteration step.

The radii of the disks, necessary for evaluating the Minkowski dimension, were approximated by squares with the length (2*n*+1) with *n* = 0,1,2,…,20. The corresponding radii *r* are given by:



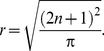
(7)Giving the fact that natural structures represented in digital images are of course not perfect fractals but show fractal properties in certain range of scales. A linear relationship between the scale of measurement and object size is therefore not guaranteed over the whole range. The values of ε and r included in the linear regression analysis are crucial since they determine the absolute value of the fractal dimension and have to be chosen carefully. For each individual regression, the correlation coefficient R was calculated starting with the smallest possible value for ε_min_ and a value slightly above was chosen for ε_max_. Subsequently ε_max_ was increased until R did not change significantly giving the maximum value of ε. In a second step ε_min_ was increased until R did not change significantly providing the minimum value of ε. With that, we defined a correlation coefficient of R = 0.995 for the linear regression as a threshold for including data points which led to a range of n = [1], [8] for the Box-counting dimension and n = [6], [20] for the Minkowski dimension. Both methods were applied for the whole skeletonized image and separately for the left and right hemisphere using software built in house (http://code.google.com/p/iqm/downloads/detail?name=Iqm_1.12.001.exe).

Multifractal analysis was carried out using FracLac (Karperien, A., FracLac for ImageJ, version 2.5; http://imagej.nih.gov/ij/plugins/fraclac/fraclac.html), a plugin for ImageJ software (Wayne Rasband, National Institutes of Health, USA). Generalized dimensions were evaluated within the range q = [0,5] using a box size range of n = [1], [8].

### Simulation

The influence of the image matrix size on *FD* was investigated using a statistical fractal obtained by diffusion limited aggregation [35]. DLA is the process when particles, which perform a random walk due to Brownian motion, aggregate and form a cluster. Ten fractals were generated and *FD* (*D_b_*, *D_m_*) was calculated for the whole image and separately for the left and right half of the images. This was carried out for DLA fractals with a matrix size of 512×512 pixels and of 364×436 pixels, which is equivalent to the image size of in vivo data, and 256×512 pixels and 183×436 pixels respectively.

### Statistical Analysis

The group of ten patients (P) was divided into two subgroups including hemispheres with AVM (P_AVM_) and hemispheres without AVM (P_no AVM_). Subsequently, from the group of healthy controls (HC) two subgroups were built containing the left and the right hemisphere (HC_left_, HC_right_). The significances of the differences in *FD* values of these groups were quantified using a 2×2 mixed-design ANOVA test with a within-subjects factor of hemisphere (for patients: AVM, no AVM, for healthy controls: left, right) and a between-subject factor of group (P, HC) for both methods (*D_b_*, *D_m_*) evaluating *FD*. Statistical differences in *FD* for the whole images of patients and controls (P_total_, HC_total_) were tested using an unpaired, two-tailed t-test. Results from the multifractal analysis were analyzed using a total of three paired, two-tailed t-tests to investigate statistical differences between the hemisphere with AVM and the hemisphere without AVM for *D_0_*, *D_1_* and *D_2_*. Multifractality was investigated using a total of four paired, two-tailed t-tests to test the significance between *D_0_*, *D_1_* and *D_2_* for the hemisphere with AVM and the hemisphere without AVM. To ensure the applicability of these statistical tests, the normal distribution of the data was tested using the Kolmogorov-Smirnov test. The significance of differences in *FD* for ten statistical fractals with regards to image matrix size was tested using a total of four 3-way ANOVAs for repeated measurements comparing *D_b_* and *D_m_* for the total images matrix sizes 512×512 and 364×436 and their left and right image halves. The level of significance was set at p<0.05 for all tests. All statistical analysis were carried out using the statistical package IBM SPSS Statistics 19 (SPSS Inc., Chicago, Illinois, USA).

Linear regression models were used to test for correlation first, between *FD* and the maximum slope of contrast tracer transit and second, between *FD* and the nidus size. A post hoc power analysis was performed to estimate the sample size for *D_b_*, *D_m_*. *D_0_*, *D_1_* and *D_2_* based on the correlation coefficients.

## Results

To investigate if the fractal dimension can measure vascular complexity in patients with AVM, the Box-counting dimension, the Minkowski dimension and the generalized dimensions have been calculated. To ensure that *FD* specifically captures the higher vascular complexity in AVMs, *D_b_* and *D_m_* were evaluated for both hemispheres in patients and controls separately and for the whole brain. Additionally *D_0_*, *D_1_* and *D_2_* were evaluated for both hemispheres in patients. The results of the Kolmogorov-Smirnov test revealed the normal distribution of the data and ensured the applicability of the statistical tests below.

For *D_b_* the main effects of hemisphere (F(1,18) = 6.8, p = 0.017, η = 0.07) and group (F(1,18) = 4.6, p = 0.045, η = 0.21) were qualified by an interaction between hemisphere and group (F(1,18) = 7.0, p = 0.016, η = 0.07). For *D_m_* the main effects of hemisphere (F(1,18) = 5.7, p = 0.028, η = 0.18) and group (F(1,18) = 3.5, p = 0.078, η = 0.16) were again qualified by an interaction between hemisphere and group (F(1,18) = 6.6, p = 0.019, η = 0.21).

Posthoc-analysis using Bonferroni adjustment for multiple comparison indicated that patients had significantly higher *FD* values for *D_b_* and *D_m_* in hemispheres with AVM compared with the hemispheres without AVM (*D_b_*: p = 0.002, *D_m_*: p = 0.002). For both methods healthy controls had similar values for *FD* in both hemispheres with no significant differences in *FD* (*D_b_*: p = 0.982, *D_m_*: p = 0.892). No significant differences were observed comparing the non affected hemisphere of patients with healthy controls (HC_left_: *D_b_*: p = 0.574, *D_m_*: p = 0.918, HC_right_: *D_b_*: p = 0.691, *D_m_*: p = 0.872) but significant differences when comparing the patient’s hemisphere with AVM with the hemispheres of healthy controls (HC_left_: *D_b_*: p = 0.015, *D_m_*: p = 0.020, HC_right_: *D_b_*: p = 0.010, *D_m_*: p = 0.024).

**Figure 2:**
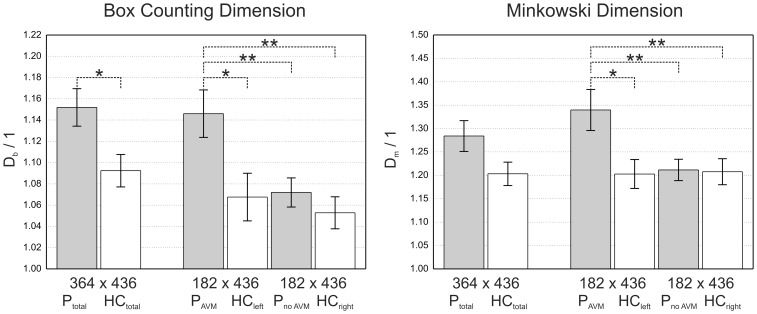
Mean values and standard error mean of *D_b_* (left side of the figure) and *D_m_* (right side of the figure) comparing patients (P_total_) with healthy controls (HC_total_) for the whole image, hemispheres of patients with AVM (PAVM) compared to the hemisphere without AVM (_Pno AVM_) and comparison of the left and right hemisphere of healthy controls (HC_left_, HC_right_). Asterisks indicate significant differences in group comparisons: * p<0.05, ** p<0.01.

Comparing patients with controls for the whole image showed a significantly higher value of *D_b_* (p = 0.020) and a statistical trend for elevated *D_m_* (p = 0.067) in patients. Overall *D_m_* was higher than *D_b_* for all subgroups in patients and controls. Mean values and standard error mean for all groups are reported in Table 1 and summarized in Figure 2.

**Table 1:** Mean values of *D_b_* and *D_m_* for the patients’ hemispheres with AVM (P_AVM_), hemispheres without AVM (P_no AVM_) and the entire brain (P_total_).

		*D_b_*		*D_m_*	
		Mean	SEM	Mean	SEM
Patients	P_AVM_	1.146	0.022	1.340	0.044
	P_no AVM_	1.072	0.014	1.212	0.023
	P_total_	1.152	0.018	1.284	0.033
Healthy Controls	HC_left_	1.068	0.022	1.203	0.031
	HC_right_	1.053	0.015	1.208	0.028
	HC_total_	1.092	0.015	1.203	0.025
DLA	256x512_left_	1.392	0.007	1.595	0.009
	256x512_right_	1.390	0.008	1.601	0.010
	512x512	1.390	0.005	1.597	0.007
	182x436_left_	1.387	0.009	1.591	0.011
	182x436_right_	1.297	0.008	1.580	0.009
	364x436	1.360	0.005	1.587	0.005

Mean values of *D_b_* and *D_m_* for healthy controls including the left and right hemispheres (HC_left_, HC_right_) and the entire brain (HC_total_). Mean values of *D_b_* and *D_m_* are presented for ten statistical fractals obtained by DLA for images different image matrix sizes. Values are as mean ± standard error of the mean (SEM).

**Figure 3:**
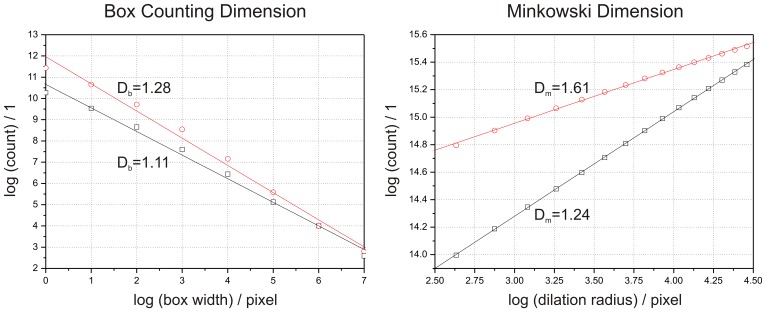
The curves in the double logarithmic plots show the relationship between the scales of measurement and object size in one representative patient. Values of *FD* assessed by means of *D_b_* (left side of the figure) and *D_m_* (right side of the figure) were evaluated from the different slopes using Eq. (3) and (4). The red lines were obtained from the hemisphere with AVM and the black lines were obtained from the hemisphere without AVM demonstrating that *FD* is sensitive to the vascular complexity due to AVMs.

To illustrate the proposed methods, the log-log plots for *D_b_* and *D_m_* are shown in Figure 3 for a single patient. The different slopes reveal the higher *FD* in the left hemisphere suffering from a large AVM (nidus size = 2.9 cm) (*D_b_* = 1.28, *D_m_* = 1.61) compared to the unaffected hemisphere (*D_b_* = 1.11, *D_m_* = 1.24).

**Figure 4:**
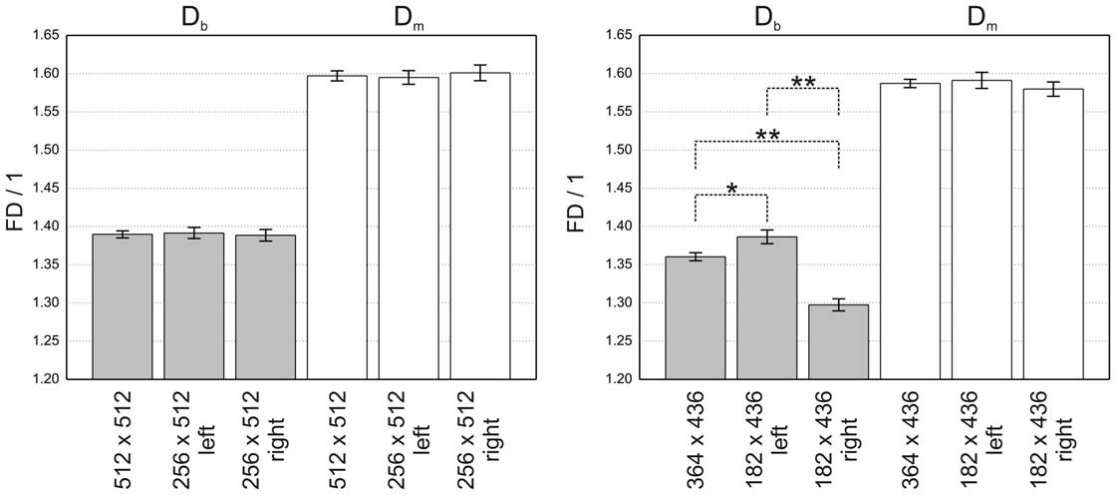
Mean values and standard error mean of *D_b_* and *D_m_* for statistical fractals obtained by DLA. On the left side of the figure fractals with an image matrix size of 512×512 pixels and 256×512 pixels for the image halves were analyzed. On the right side of the figure fractal analysis based on image matrix size of 364×436 pixels and 182×436 pixels for the image halves showing significant differences in *D_b_* due to image matrix size. Asterisks indicate significant differences in group comparisons: * p<0.05, ** p<0.01.

*FD* of ten statistical fractals obtained by DLA was analyzed to study the influence of the image matrix size on the quantitative results. *D_b_* and *D_m_* were evaluated for the image matrix size of 512×512 pixels and 256×512 pixels for the left and right image halves respectively. No significant differences were found between different image matrix sizes for this resolution within one method. Subsequently, *D_b_* and *D_m_* were evaluated for an image matrix size which matches the matrix size of the in-vivo data of 364×436 pixels and 182×436 pixels for the left and right halves respectively. Only the ANOVA for *D_b_* yielded a significant difference (F(1,18) = 60.104, p = 0.000, η = 0.875) between the whole image and both image halves. No significant differences were found between different matrix sizes for this resolution using the Minkowski method. These results reveal that *FD* evaluated by the Box-counting method is sensitive to the image matrix size. Mean values and standard error mean for all groups are reported in Table 1 and summarized in Figure 4.

**Figure 5:**
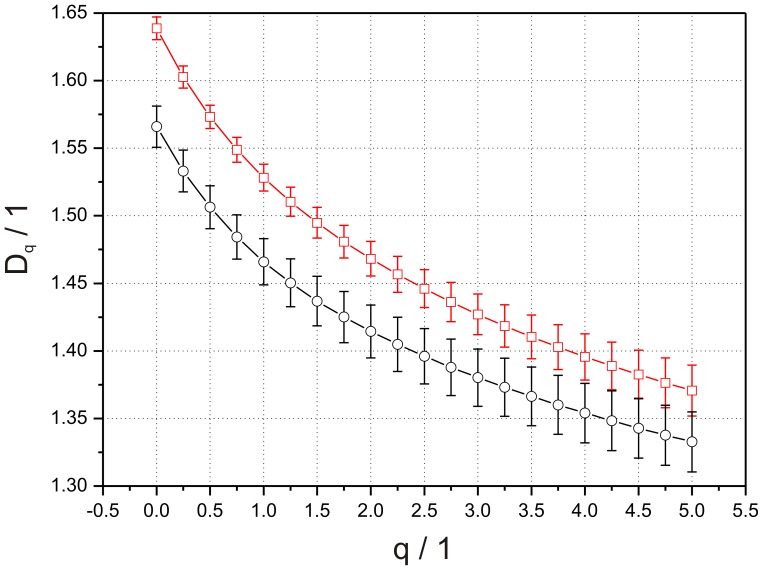
Mean values and standard error mean of the generalized dimensions in the range q = [0,5] obtained by multifractal analysis. The red line indicates the patient’s hemisphere with AVM, the black line the hemisphere without AVM.

**Figure 6:**
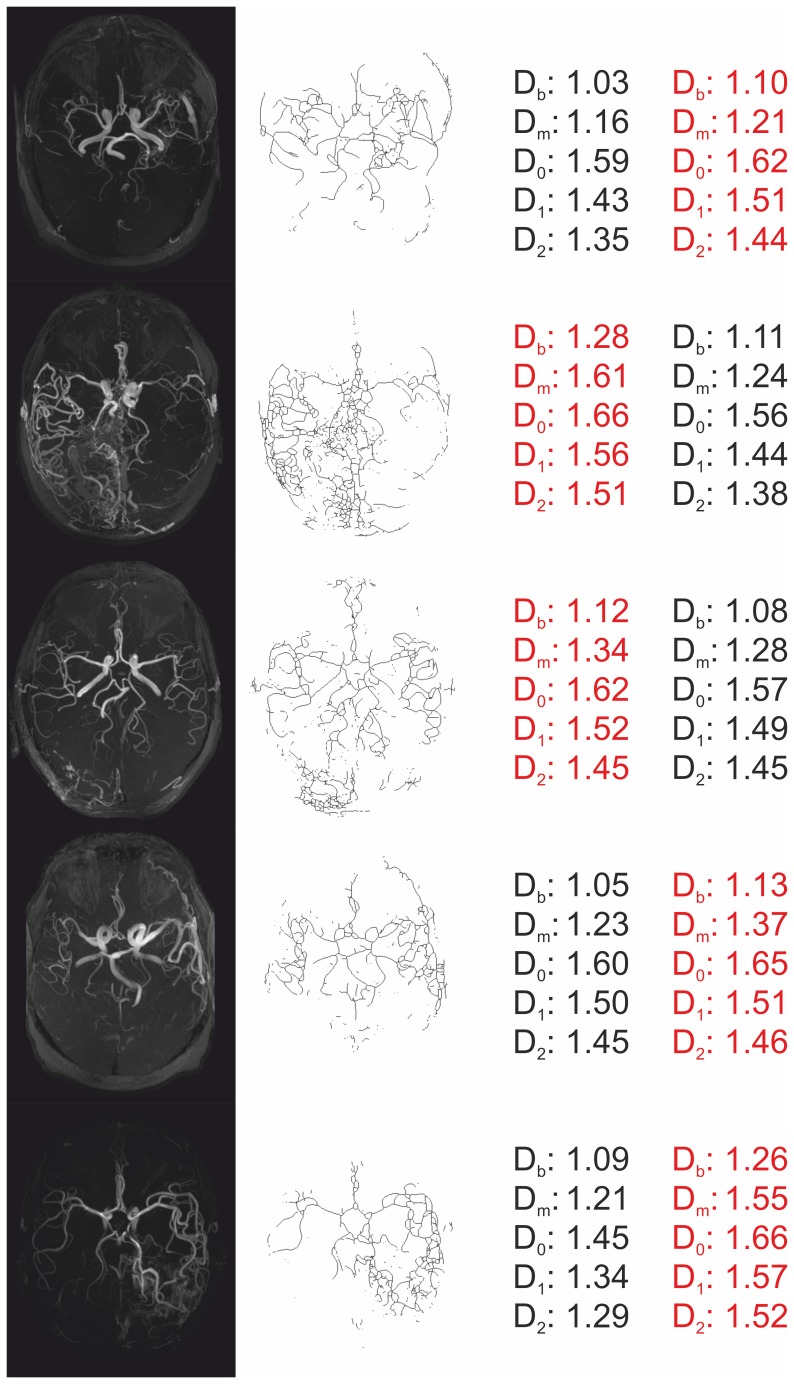
MIP images of five patients (first column), skeletonized images (second column) and values of FD for *D_b_*, *D_m_*, *D_0_*, *D_1_*, and *D_2_* for the left and the right hemispheres (red indicates the hemisphere with AVM).

The generalized dimensions were higher in the hemisphere with AVM compared to the hemisphere without AVM for all q in the evaluated range (Figure 5). Significantly higher *FD* values were found for *D_0_*, *D_1_*, and *D_2_* in hemispheres with AVM compared with the hemispheres without AVM (*D_0_*: p<0.001, *D_1_*: p<0.001, *D_2_*: p = 0.036). Mean values and standard error mean for *D_0_*, *D_1_*, and *D_2_* are reported in Table 2. Differences between *D_0_*, *D_1_*, and *D_2_* were significant in both hemispheres with and without AVM (all p<0.001) indicating that the cerebral vascular system show multifractal properties. Examples for five patients are shown in Figure 6 providing MIP images, skeletonized images and values of FD (*D_b_*, *D_m_, D_0_*, *D_1_*, and *D_2_*) for the left and the right hemispheres.

**Table 2:** Mean values of *D_0_*, *D_1_* and *D_2_* for the patients’ hemispheres with AVM (P_AVM_), hemispheres without AVM (P_no AVM_) and the entire brain (P_total_).

		*D_0_*		*D_1_*		*D_2_*	
		Mean	SEM	Mean	SEM	Mean	SEM
Patients	P_AVM_	1.639	0.008	1.528	0.010	1.468	0.017
	P_no AVM_	1.566	0.015	1.466	0.017	1.414	0.020
	P_total_	1.569	0.007	1.496	0.012	1.458	0.013

Values are as mean ± standard error of the mean (SEM).

**Figure 7:**
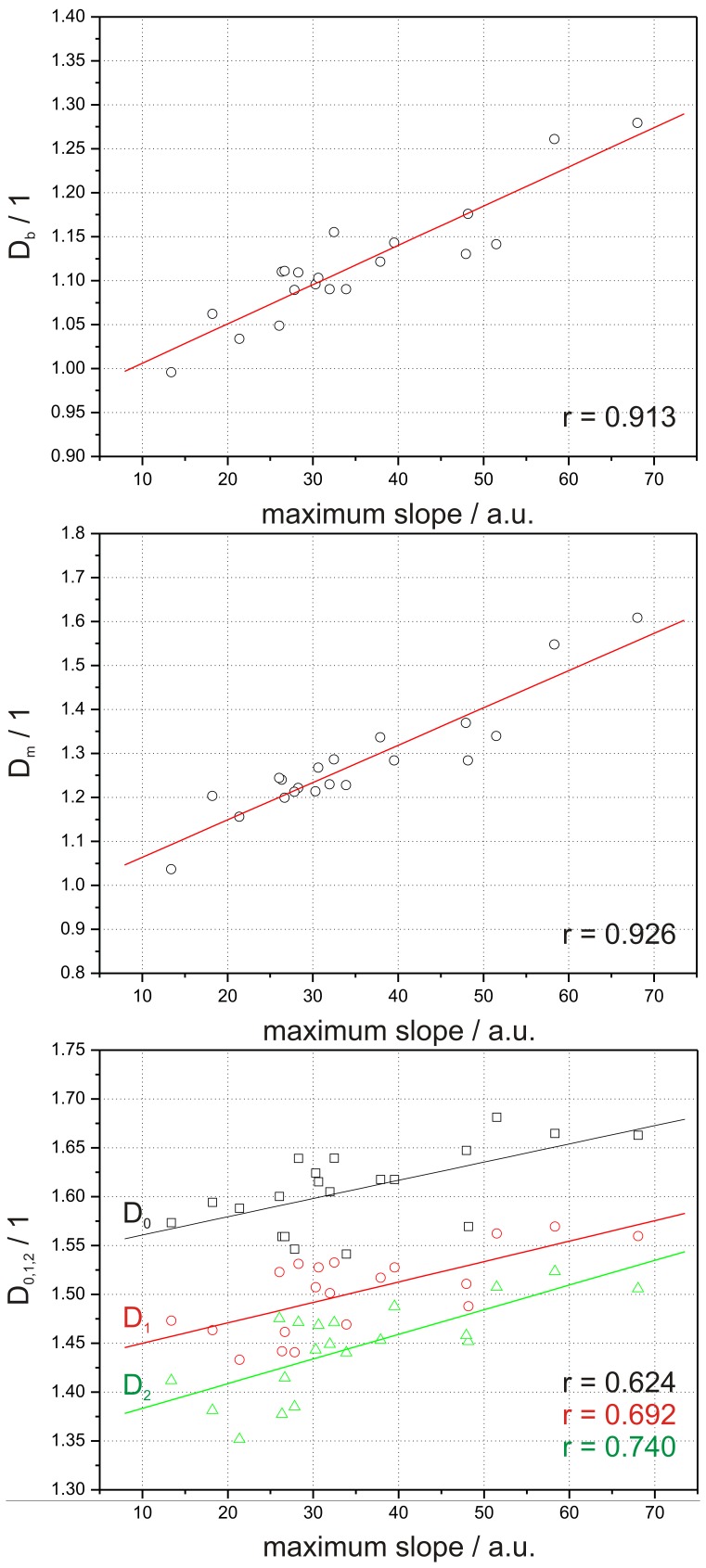
Correlation between values of *D_b_*, *D_m_*, *D_0_*, *D_1_*, and *D_2_* and the maximum slope of contrast media transit obtained from DCE-MRI data.

**Figure 8:**
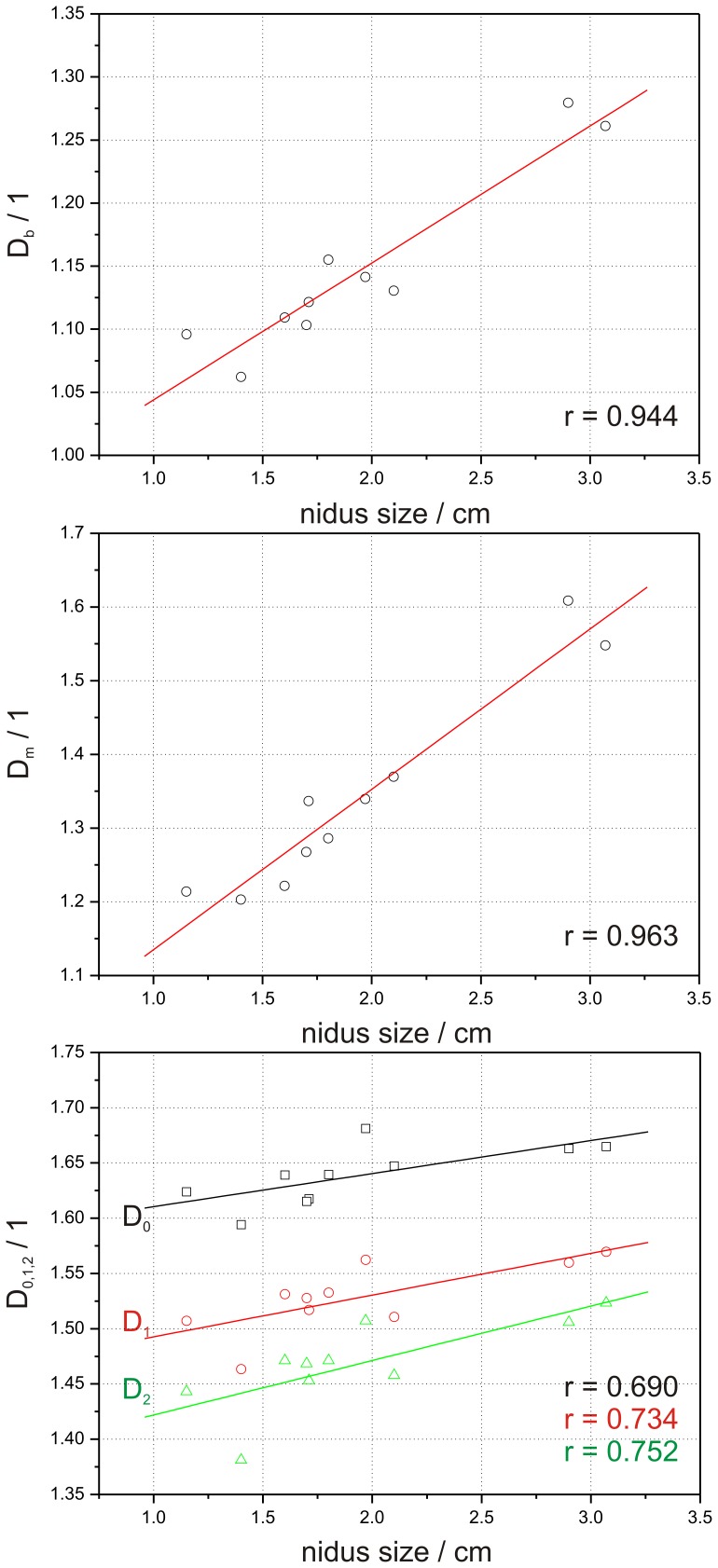
Correlation between values of *D_b_*, *D_m_*, *D_0_*, *D_1_*, and *D_2_* and the nidus size evaluated from X-ray angiography data.

For the observed correlations between *FD* and the contrast media transit the post hoc power analysis estimated a sample size of n = 7 for *D_b_*, n = 7 for *D_m_*, n = 23 for *D_0_*, n = 17 for *D_1_* and n = 14 for *D_2_*. The sample size for the correlation between *FD* and nidus size was estimated as follows: n = 7 for *D_b_*, n = 6 for *D_m_*, n = 25 for *D_0_*, n = 20 for *D_1_* and n = 19 for *D_2_*. The linear regression analysis showed a strong and positive linear correlation between *FD* of the affected hemisphere and the maximum slope of contrast media transit for *D_b_* and *D_m_* (*D_b_*: r = 0.913, p<0.0001; *D_m_*: r = 0.926, p<0.0001), (Figure 7). A strong correlation was also found between *FD* and the nidus size (*D_b_*: r = 0.944, p<0.0001; *D_m_*: r = 0.963, p<0.0001), (Figure 8). Overall *D_0_*, *D_1_*, and *D_2_* showed a weaker positive linear correlation compared with *D_b_* and *D_m_* for both the maximum slope of contrast media transit (*D_0_*: r = 0.624, p = 0.003; *D_1_*: r = 0.692, p<0.001; *D_2_*: r = 0.740, p<0.001), (Figure 7) and the nidus size (*D_0_*: r = 0.690, p<0.027; *D_1_*: r = 0.734, p<0.016; *D_2_*: r = 0.752, p<0.012), (Figure 8).

## Discussion

Fractal analysis has been shown to provide useful information in analyzing natural structures by means of their space filling properties in many applications in biology and medicine [12], [15], [17], [22]. Giving the fact that the structures to be analyzed are usually represented in digital images with a certain resolution defined by the image matrix, scales and order of magnitudes are always restricted. In order to apply fractal analysis to digital images it has to be assured that the scaling range covers at least two orders of magnitude [8] providing an estimation of the fractal dimension. Since this criterion was met for our data, we showed as a proof of concept that *FD*, a measure for the fractal nature of complex structures, is significantly elevated in cerebral vascular systems with higher structural complexity in AVMs and that *FD* strongly correlates with physiological and anatomical properties like vascular flow and nidus size. This new method may thus provide an objective and sensitive measurement for structural vascular complexity with some belongs on important characteristics of AVMs.

Two methods, the Box-counting method and the Minkowski method were applied to access *FD* in ten patients with AVM and ten healthy controls. Additionally multfractal analysis was performed for patients to study multifractal properties of the cerebral vascular system. The interpretation of a spectrum of generalized dimensions is not trivial especially in the light of correlation with physiological parameters. It has been shown that the spectrum of generalized dimensions is shifted due to pathologic changes [42]. Giving the fact that such variation in the spectrum can not be described with a single parameter that can be correlated with physiological parameters, we focused on the separate evaluation of three specific dimensions (*D_0_*, *D_1_*, and *D_2_*) as a subset of the generalized dimension spectrum. This justifies that the generalized dimensions were evaluated in the range q = [0,5] supported by several concerns about the interpretation of generalized dimensions for q<0 [43], [44].

Our strategy to evaluate *FD* separately for both hemispheres and the whole brain ensured that higher *FD* values could be associated specifically with vessel complexity due to AVMs despite anatomical issues. All methods showed significantly higher *FD* in the hemisphere with AVM compared to the hemisphere without AVM. While *D_b_*, evaluated for the whole brain was significantly higher in patients than in controls, *D_m_* showed a statistical trend for higher values. Considering the variability of *FD* between the subjects this effect can be explained by the limited number of subjects involved in this study. In patients *D_b_* evaluated for the whole brain was higher than *D_b_* evaluated for the hemisphere with AVM. This finding supports the fact, that absolute values for different image sizes are not comparable using the standard Box-counting method. No such effect could be observed for the dimensions *D_m_*, *D_0_*, *D_1_*, and *D_2_*. We also found, that *D_0_*, *D_1_*, and *D_2_* were significantly different fulfilling equation (6). This may indicate that the cerebral vascular system shows multifractal properties.

As would be expected, *FD* strongly correlates with the maximum slope of contrast media transit because the tracer transit time is related to the number of vessels feeding the nidus. The strong correlation between *FD* and the nidus size is not that obvious but suggests that there is a relation between vascular complexity and nidus size. *D_0_*, *D_1_*, and *D_2_* obtained from the multifractal analysis show a weaker correlation with the tracer transit time and the nidus size compared with *D_b_* and *D_m_*. This observation suggests that *D_b_* and *D_m_* are more robust parameters for characterizing physiological properties in patients with AVM. One reason might be the sensitivity of MFA with regards to object size as pointed out in [33], [45]. Taking into account that the post hoc power analysis estimated a larger sample size required for the correlation between *D_0_* and the tracer transit time and for *D_0_*, *D_1_*, and *D_2_* and the nidus size, correlation results from the MFA can not be considered as reliable. However more cases have to be analyzed to proof if MFA provide information superior to fractal analysis.

Many methods with different theoretic assumptions have been developed to evaluate *FD* [10]. *D_b_* is the most prominent method to obtain *FD* because of its simplicity. However, this method also has some limitations in that absolute values are sensitive with regards to image size [46] defined by the image matrix. Giving the fact that the box size is doubled at each iteration step starting with the size of one pixel, a complete covering of the image is only possible if the dimensions of the image to be analyzed are given as a power of two. If this is not the case, *FD* is dependent from the initial location of the grid. For our data, this resulted in different values of *FD* for the whole brain and the two hemispheres. Ten statistical fractals obtained by DLA showed that *D_b_* of the whole image equals *D_b_* of the halves using a matrix of 512×512 pixels and 256×512 pixels respectively. Values of *D_b_* significantly differed when using a matrix of 364×436 pixels for the whole image and 182×436 pixels for the two halves. This observation was also confirmed for in vivo data from healthy controls. Even though this effect was small compared to differences in *D_b_* due to AVMs it can not be neglected. To overcome these limitations in the Box-counting method extensions of this technique have been proposed [10] such as the sliding Box-counting dimension in which each box is slid over the image overlapping the previous box. However, the assessment of *FD* by evaluating the Minkowski dimension uses a different concept in which the structure itself is covered through geometrical objects such as circles or triangles. This strategy reduces variations in *FD* due to different image matrix sizes as we demonstrated in this paper. No significant differences in *D_m_* could be observed for different image matrix sizes neither in computer simulations nor for in vivo data. These results suggest that the Minkowski dimension is a suitable measure for analyzing images with image dimensions not based on 2^n^.

Even though all methods *D_b_*, *D_m_* and the generalized dimensions are measurements for *FD*, differences between these methods could be observed for our data. Overall the generalized dimensions showed the highest absolute values. *D_m_* was higher than *D_b_* for all subgroups. *D_b_* and *D_m_* are both approximations of the Hausdorff dimension which are only equal for strictly self similar objects [47], [48]. This is not fulfilled for biological structures explaining the observed differences. It has to be noted, that *FD* values obtained by different algorithms vary in a wide range and that absolute values are hardly comparable [49]. Many parameters such as the starting point of the initial grid in the Box-counting dimension, the margin around the object and the number of data points involved in the regression analysis affect the computation of absolute values of *FD*. Specifically in the simulation analyzing a fractal obtained by DLA, the Box-counting dimension shows a value of around 1.39. This can be explained through the fact that *D_b_* is prone to margins without any texture since *D_b_* is sensitive to the overall complexity. The Minkowski dimension measures the local connectivity of the object and calculates an average value making this method less sensitive to this border effects. Our values of *FD* for the DLA are around 1.6 for the Minkowski method and are inline with values reported in the literature.

As expected, the log-log plots representing the scaling characteristics of the vascular system did not show a linear relationship between the scale of measurement and the object size. Hence, the slope of the log-log plot would provide biased values of *FD* when using all data points for the linear regression. Complex methods have been developed, trying to identify the largest range of self-similarity. These techniques are based on combination of curve-fitting tests and curvilinearity tests [50]. One way to address this issue is to use the correlation coefficient as a threshold for determining the range of linearity. We used this method in our work resulting in a constant number of data points for which the linear regression was carried out in all subjects.

Although it is possible to evaluate *FD* from gray scale images, in this study all methods for evaluating *FD* were applied on binary images. For the evaluation of vascular complexity the relevant information is the branching pattern, the side length of individual branches and the angles between them. Hence, gray values in MIP images do not provide additional interpretable information the evaluation of *FD* from binary images seems reasonable. To obtain binary images from TOF MIP images several preprocessing steps are involved. These steps include vessel segmentation and skeletonizing. Many methods have been developed and applied for segmenting the vascular tree [51]. In this work we used a k-means clustering algorithm for segmentation and the skeletonize algorithm implemented in the ImageJ software. Our strategy provided binary images that sufficiently captured small vessels in AVMs and worked reliably for all data. This was confirmed by a visual comparison to ensure that even small arteries feeding the nidus and are visible in the MIP images are represented in the skeletionized images. However, the choice of the segmentation algorithm is crucial for the absolute values of *FD* and parameters influencing the preprocessing steps must be strictly kept constant for group comparisons.

AVM is a cerebral vascular disease, which is heterogeneous in its appearance. The more ore less compact nidus can be supplied by one or more feeding arteries, shunting blood to one ore more draining veins. Using the proposed method, specifically the structural complexity of the feeding arteries has an influence on *FD*. The investigation of AVM by means of MRI usually includes dynamic sequences providing hemodynamic information about the vascular system. This information is an important additional information helping neuroradiologists to classify the AVM. Even though the evaluation of *FD* does not include any information about the vascular hemodynamic hence only anatomical structures are evaluated with regards to their complexity, the blood flow is indirectly reflected in *FD* due to the increased number of vessels.

It has to be noted that a MIP of 3D-TOF MR images is only an approximate representation of the vascular system giving the fact that superimposed vessels can not be discriminated for a fixed projection plane. Ideally a three dimensional structure should be analyzed by three-dimensional fractal analysis providing *FD* values between two and three. The projection of a fractal from a m-dimensional space into a (m-1) dimensional subspace is well defined [36] and explain our values of *FD* between one and two. However, a three-dimensional fractal analysis requires an accurate 3D model of the structure to be analyzed. While in the field of neuroimaging this technique has successfully been applied for the analysis of a 3D gray matter model extracted from T_1_ weighted MR images [24], a 3D model from the cerebral vascular system from TOF-MRI can not be obtained easily. Even though 3D vessel segmentation algorithms are explored intensively in medical image analysis [52], the algorithms are optimized for a specific imaging modality and can not be easily applied on TOF-MRI data due to low contrast and limited resolution. Segmentation methods that specifically deal with TOF-MRI data [53] are relative new, complex and clinically not verified. Hence they are not “state-of-the-art” and not included in image processing tools provided by MR manufactures. On the other hand MIP is available on every scanner and is a standard representation of TOF-MR images commonly used by clinicians. Since the focus of this work is on the clinical applicability of the proposed method and considering the strong correlation between *FD* and physiological parameters the approximation we used seems to be reasonable. Nevertheless, the 2D fractal analysis is a limitation in the proposed method especially in the light of providing absolute values of FD from the 3D vascular structure. However, more studies have to be made to investigate 3D vessel segmentation algorithms based on TOF-MRI data focusing on vascular diseases such as AVM. These future studies may provide the basis for the application of 3D fractal analysis on the human cerebral vascular system.

In principle, our proposed method can also be applied to MIP images obtained by contrast enhanced MRA. Though this technique provides a good representation of the vascular system, it inherits the problem of contrast agent bolus timing. The time between the application of the contrast agent and the image acquisition heavily influences the weighting of the arterial system and the venous system. As it is difficult to keep this weighting constant for all patients we evaluated images based on a native TOF sequence. However, the applicability of this technique is not limited to any special sequence if the image resolution is high enough.

### Conclusions

In this work we present a novel approach for the quantitative measure of cerebral vascular complexity by means of fractal dimension. Our results suggest that *FD* assessed by the Box-counting dimension and the Minkowski dimension is related to structural vascular complexity due to the increased number of feeding arteries in patients suffering from AVM. The strong correlation between *FD* and the maximum slope of contrast media transit and between *FD* and the nidus size, as determined by the gold-standard X-ray angiography, underlines the physiological relevance of this measure. *FD* analysis is a simple and robust technique that may yield an objective measure for investigating vascular disorders such as AVM. However, further studies are needed including more patients to determine the sensitivity of the proposed methods.
